# The aberrant asynchronous replication — characterizing lymphocytes of cancer patients — is erased following stem cell transplantation

**DOI:** 10.1186/1471-2407-10-230

**Published:** 2010-05-24

**Authors:** Arnon Nagler, Samuel Cytron, Maya Mashevich, Avital Korenstein-Ilan, Lydia Avivi

**Affiliations:** 1 Bone Marrow Transplantation Department, Institute of Hematology, Chaim Sheba Medical Center, Tel-Hashomer 52621, Israel; 2 Department of Urology, Barzilai Medical Center, affiliated to the Faculty of Health Sciences, Ben-Gurion University of The Negev, Askelon 78306, Israel; 3 Department of Human Molecular Genetics and Biochemistry, Sackler School of Medicine, Tel-Aviv University, Tel-Aviv 69978, Israel

## Abstract

**Background:**

Aberrations of allelic replication timing are epigenetic markers observed in peripheral blood cells of cancer patients. The aberrant markers are non-cancer-type-specific and are accompanied by increased levels of sporadic aneuploidy. The study aimed at following the epigenetic markers and aneuploidy levels in cells of patients with haematological malignancies from diagnosis to full remission, as achieved by allogeneic stem cell transplantation (alloSCT).

**Methods:**

*TP53 *(a tumor suppressor gene assigned to chromosome 17), *AML1 *(a gene assigned to chromosome 21 and involved in the leukaemia-abundant 8;21 translocation) and the pericentomeric satellite sequence of chromosome 17 (*CEN17*) were used for replication timing assessments. Aneuploidy was monitored by enumerating the copy numbers of chromosomes 17 and 21. Replication timing and aneuploidy were detected cytogenetically using fluorescence *in situ *hybridization (FISH) technology applied to phytohemagglutinin (PHA)-stimulated lymphocytes.

**Results:**

We show that aberrant epigenetic markers are detected in patients with hematological malignancies from the time of diagnosis through to when they are scheduled to undergo alloSCT. These aberrations are unaffected by the clinical status of the disease and are displayed both during accelerated stages as well as in remission. Yet, these markers are eradicated completely following stem cell transplantation. In contrast, the increased levels of aneuploidy (irreversible genetic alterations) displayed in blood lymphocytes at various stages of disease are not eliminated following transplantation. However, they do not elevate and remain unchanged (stable state). A demethylating anti-cancer drug, 5-azacytidine, applied in vitro to lymphocytes of patients prior to transplantation mimics the effect of transplantation: the epigenetic aberrations disappear while aneuploidy stays unchanged.

**Conclusions:**

The reversible nature of the replication aberrations may serve as potential epigenetic blood markers for evaluating the success of transplant or other treatments and for long-term follow up of the patients who have overcome a hematological malignancy.

## Background

Genes of the parental genomes in diploid organisms usually maintain functional symmetry, namely, the two alleles operate or shut off concomitantly in what is called biallelic expression. However, a subset of genes exhibits allele-specific expression (monoallelic expression) in which only one allele operates and its counterpart remains silent [1,2]. The determination of whether or not allelic counterparts of a gene retain functional symmetry is usually fixed at early stages of development and is an essential aspect of a usually heritable epigenomic contour passed down to the cell's progeny (reviewed in [2]).

Whatever the mechanisms involved in the maintenance and selection of an allele for an allele-specific epigenetic profile, it is usually accompanied by the two alleles maintaining different chromatin structures and/or differential methylation capacities in which asynchronous DNA replication plays a decisive role (reviewed in [3]). Specifically, an asynchronous pattern of allelic replication (in contrast to a highly synchronous pattern characterizing alleles of biallelically expressed genes) was shown for all known types of monoallelically expressed genes and not necessarily in the tissue of expression. These include (i) imprinted genes [4-8]; (ii) genes subjected to X-chromosome inactivation [9,10]; and (iii) genes undergoing allelic exclusion [11-13].

The fluorescence in situ hybridization (FISH) replication assay is the method of choice for evaluating the temporal order of allelic replication [11-14]. Applying the FISH replication assay to biallelically expressed genes in cells of cancer patients indicated that malignancy is associated with non-disease-specific loss of the normally synchronous pattern of replication of certain loci. Briefly, biallelically expressed genes, -- such as *TP53*, *AML1*, *RB1, C-MYC *and *HER2 *(which normally display a synchronous mode of replication) -- exhibit an asynchronous pattern of replication (similar to that of monoallelically expressed genes) in bone marrow cells [15] and blood lymphocytes [16] of patients with hematological malignancies, and even in blood lymphocytes of patients with solid tumors, such as renal cell carcinoma [7] or prostate cancer [8,17].

Furthermore, chromosome-specific sequences (pericentromeric, unexpressed DNA arrays), which normally display synchrony in replication of homologous counterparts, similar to biallelically expressed genes, change their inherent replication mode and replicate asynchronously in lymphocytes of patients with cancer, including ovarian [18], hematological [16] and prostate [8,17] malignancies. This change in the replication patterns of the pericentromeric arrays is accompanied by chromosomal malsegregation, which results in increased aneuploidy in the patients' cells [8,16,18].

The cancer-mediated loss of synchronous replication appears to be a reversible epigenetic phenomenon associated with abnormal methylation capacity since it was reversed to the normal following in vitro application of the methylation inhibiting drug 5-azacytidine (AZA). This demethylating agent is known to induce the re-expression of epigenetically silenced genes [19] and has recently been approved for the therapy of myeloid malignancies [20]. The readjusted synchronous replication in the presence of AZA was demonstrated in blood lymphocytes of cancer patients [8,16,17]. In contrast, the increased aneuploidy in the lymphocytes of the patients remains unchanged following AZA application [8,16].

Interestingly, the short term effect of granulocyte colony-stimulating factor (G-CSF), a key haematopoietic growth factor, used clinically, in healthy individuals (siblings as well as unrelated stem cell donors) to obtain cells for allogeneic transplantations, resembles, in this respect, the effects of malignant disease on blood cells. Briefly, G-CSF applied to normal healthy individuals, in vivo or in vitro, leads to a loss of synchronous replication of biallelically expressed genes in their blood lymphocytes, which is eliminated in vitro with AZA [21]. G-CSF concomitantly causes increased aneuploidy, which remains unchanged following in vitro application of the demethylating drug. Yet, the long-term effect of G-CSF following in vivo application to healthy stem cell donors resembles the effect of AZA applied in vitro to cells of cancer patients -- the aberrant replication is set to normal whereas the increased aneuploidy remains unchanged [21].

The present study was carried out to follow gene replication patterns (epigenetic properties) and aneuploidy levels (genetic properties) in patients' lymphocytes prior to and following successful allogeneic stem cell transplantation (alloSCT). Specifically, we show here that (i) the loss of synchronous replication of loci that normally replicate synchronously, in the patients' cells prior to alloSCT is set to normal following transplantation, while the increased aneuploidy remains abnormal; and (ii) AZA applied in vitro to lymphocytes of patients prior to alloSCT mimics the effect of alloSCT -- the aberrant epigenetic profiles observed in lymphocytes of patients prior to alloSCT are set to normal, whereas the accompanying genetic aberrations are unaffected.

## Methods

### Peripheral Blood Samples

Sixty-four peripheral blood samples (5 ml, each) obtained from four groups of consenting individuals were investigated: (i) 20 from normal, cancer-free individuals, used as controls (hereafter termed CON); (ii) 20 from patients with hematological malignancies taken at diagnosis (hereafter termed ADI); (iii) 14 of patients with hematological malignancies obtained prior to allogeneic stem cell transplantation (alloSCT) (hereafter termed PRE); and (iv) 10 from patients with hematological malignancies taken following alloSCT, each displaying 100% donor chimerism, as judged from analyses of bone-marrow aspirates (hereafter termed POST). None of the investigated individuals was included in more than one group. The subjects of each group were chosen at random in order to avoid any bias in patients' selection.

The patients suffered from various hematological malignancies. Of the 20 ADI individuals 9 had acute myelogenous leukemia (AML), 8 chronic myelogenous leukemia (CML), 2 acute lymphoblastic leukemia (ALL), and 1 multiple myeloma (MM). Of the 14 PRE individuals, 4 had AML (3 in remission and one not), 8 CML (6 in the chronic and 2 in the accelerated phase), 1 ALL (in remission) and 1 non-Hodgkins lymphoma (NHL, not in remission). Of the POST subjects, 4 had AML, 4 had CML, 1 ALL and 1 NHL (all at full remission).

The ADI group included 9 females and 11 males, ranging in age from 3 to 80 years (median and mean values of 35.0 and 37.2 years, respectively). The PRE group contained 5 females and 9 males, ranging from 11 to 56 years (median and mean of 37.5 and 37.0 years, respectively). The POST group included 3 females and 7 males, ranging from 13-51 years (median and mean of 32.0 and 32.3 years, respectively). The CON group consisted of 8 females and 12 males, ranging from 11-65 years (median and mean of 36.0 and 35.0 years, respectively).

In addition, we examined 14 peripheral blood samples (5 ml each) obtained from 14 consenting urological patients, referred to prostate biopsy because of suspected prostate cancer. This group of male patients ranged in age 53-78 years, with median and mean values of 66.5 and 65.1 years, respectively. Following biopsy, four out of the 14 patients were diagnosed with prostate cancer and the rest (10 individuals) were found to be cancer-free. Of the 10 cancer-free individuals three were found to suffer from chronic prostate inflammation and seven from benign prostate hyperplasia.

### Bone Marrow Samples

Twenty-six bone marrow samples, obtained from consenting subjects (2 ml each), were also tested. They included: (i) 12 exemplars from a new group of cancer-free individuals, 4 females and 8 males, ranging in age from 7-54 years; and (ii) 14 from patients in the PRE group, one sample from each of them.

### Cell Cultures

Cell cultures were set up according to the standard protocol for karyotype analysis [18]. Briefly, aliquots of peripheral blood or bone marrow aspirates were introduced into F-10 medium supplemented with 20% fetal calf serum, 0.2% heparin and 1% penicillin/streptomycin antibiotic solution (Biological Industries, Israel). For blood cell culturing, this medium was additionally supplemented by 3% phytohemagglutinin (PHA) to stimulate cell division. Each of the blood samples derived from the CON, ADI and PRE patients were incubated in duplicate, one in the enhanced F-10 medium containing PHA and the second in a medium containing 10^-7 ^M 5-azacytidine (AZA; Sigma, USA) as well as PHA.

After 72 hours at 37°C, colchicine (Sigma, USA) was added (to a final concentration of 5 × 10^-7 ^M) for one hr, followed by a hypotonic treatment (0.075 M KCl at 37°C, for 15 min) and four washes, each with a fresh, cold (-20°C) 3:1 methanol:acetic acid solution. The cell suspensions were stored at -20°C until used for FISH.

### Slide Preparation

The stored cell suspensions prepared for hybridization were washed twice in a 3:1 methanol: acetic acid solution, diluted until the suspension became slightly cloudy and then approximately 5 μl of the suspension was dropped onto the marked circles of two-well slide glasses. The two-well slides were obtained from Insitus Biotechnologies (Albuquerque, NM, USA) and used without any pretreatment.

### Probes

We tested four loci using directly labeled commercial probes obtained from Vysis: (i) the *TP53* probe (32-190006); (ii) the *AML1 *probe (LSI 21; 32-190002); (iii) the α-satellite probe specific for centromere 17 (32-130017, hereafter marked as *CEN17*); and (iv) the *SNRPN *probe (32-190004). The *TP53* probe identifies an archetypical tumor suppressor gene mapped to 17p13.1. The *AML1 *probe maps to chromosome 21q22, it is also used for the enumeration of chromosome 21. The centromere-specific probe *CEN17*, usually used for the enumeration of chromosome 17, identifies a specific non-coding repetitive pericentromeric array on this chromosome. The *SNRPN *probe identifies the Prader-Willi/Angelman syndrome-imprinted region on 15q11-13.

### In-Situ Hybridization

Probes were diluted in Ingen's DenHyb solutions (Insitus Biotechnologies), in catalog #D001 (1:200) for *CEN17 *and in catalog #D003 (1:100) for *AML1 *and (1:50) for *TP53 *and *SNRPN*. We used these to replace the hybridization solutions supplied with the probes. Five μl of the probe solution were placed on the target area of the sample slides and covered with a 12 mm round silanized coverslip (Insitus Biotechnologies) and sealed with rubber cement. The slides were placed on a preheated aluminum slide tray (Insitus Biotechnologies) at 76°C and denatured for 6 min at that temperature. The slide-filled aluminum slide tray was then transferred into a HybBox (Insitus Biotechnologies) covered and allowed to hybridize overnight.

### Detection

Post hybridization wash for probe *TP53 *was carried out by immersing the slides in 4 × saline sodium citrate (1× SSC = 150 mM NaCl, 15 mM sodium citrate) for five min at room temperature. Post hybridization washes for probe *AML1 *consisted of immersing the slides for 20 sec in 0.4 × SSC, pH 7.0 with 0.3% NP40 Nonidet P4 detergent), followed by 20 sec in 2.0 × SSC with 0.1% NP40 at 60°C in a shaking water bath. The post hybridization washing of the *CEN17 *was carried out in the same solutions as the *AML1 *probe, with the first wash carried out at 75°C for 2 min and the second at the same temperature for 1 min. After draining off excess liquid and brief drying, the slides were treated with 15 μl/slide of a solution of antifade containing 3 μg/ml of 4,6-diamidino-2-phenylindole (DAPI) as the counterstain (Vector Laboratories, Inc., Burlingame, CA, USA). Slides were covered with glass-coverslips (22 × 60 mm) and stored at -20°C till analyzed (which could take place anywhere between 1 hr and two days).

### Cytogenetic Evaluation

**Figure 1:**
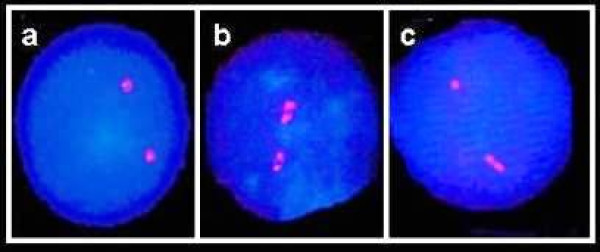
**Fluorescent signals in PHA-stimulated lymphocytes at interphase following FISH with the *CEN17 *probe**. (A) Cell with two singlets (SS cell), in which neither allele has replicated; (B) cell with two doublets (DD cell), in which both alleles have replicated; and (C) cell with one singlet and one doublet (SD cell), a S-phase cell in which one allele has replicated while its partner has still to do so.

Slides were analyzed blindly on an Olympus BH2 fluorescent microscope, using a triple band-pass filter (Chroma Technology, Brattleboro, VT, USA). The FISH replication assay was used here to follow the degree of synchrony in allelic replication (see Introduction). Accordingly, following hybridization, the fluorescence signals are divided into two categories, a single dot-like signal (designated: "singlet" or "S"), representing a yet non-replicated DNA sequence and a duplicated bipartite signal (designated: "doublet" or "D"), indicating a replicated sequence. Thus, two allelic counterparts replicating synchronously display a high frequency of cells with the two counterparts at the same replication status, either both non-replicated (SS cells; Figure 1A) or both replicated (DD cells; Figure 1B), and a low frequency of cells having one replicated and one non-replicated allele (SD cells; Figure 1C). In contrast, allelic counterparts replicating asynchronously exhibit a high frequency of SD cells. Hence, the SD cell frequency reveals the degree of asynchrony in allelic replication.

For the replication analyses, at least 100 interphase cells from each sample exhibiting two distinct well-defined fluorescence signals were scored for each treatment and probe under study. In each cell population, we noted the frequency (as a percentage, %) of SD cells out of the total population of interphase cells exhibiting two well defined fluorescence signals. We differentiated between a doublet signal and two singlets by ensuring that the two spots were not separated from each other by a distance greater than the diameter of the largest spot.

For detecting the level of aneuploidy, at least 200 interphase cells from each sample of blood preparations used for the replication analyses and treatments were screened for the numbers of chromosomes 17 and 21. For these determinations, we utilized, respectively, one-color FISH with the *CEN17 *and the *AML1 *probes. In each cell, the number of hybridization signals was recorded, and the frequency of cells with one signal was used to estimate the level of monosomic cells, the frequency of cells with three or more signals revealed the level of multisomy. Thus, the sum of the multisomy and the monosomy frequencies reflects the level of aneuploidy for the chromosome in question. The FISH interphase assay, as applied here, is currently the method of choice for detecting chromosome aneuploidy [reviewed in [21]].

### Statistical Analysis

The statistical significance of the difference between two cell populations was determined using the two-tailed Student's t-test (Microsoft Excel).

### Ethical Basis

This study was approved by the Institutional Helsinki Committee and the Israel Ministry of Health.

## Results

### The cancer-related aberrant asynchronous replication is abolished following stem cell transplantation

**Figure 2:**
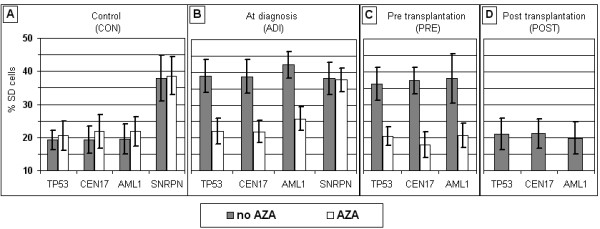
**Mean SD values in blood samples grown with and without AZA**. (A) Control samples (20 cases); (B) samples from patients with hematological malignancies obtained at diagnosis (20 cases); (C) samples from patients with malignancies taken prior to alloSCT transplantation (14 cases); and (D) samples from patients with hematological malignancies taken following alloSCT (10 cases). AZA - 5-azacytidine.

We have examined the frequency of SD cells of two genes (*TP53*, *AML1*) and a non-coding repetitive pericentromeric array (*CEN17*) in blood cell samples of patients with hematological malignancies, obtained pre and post alloSCT (PRE and POST). Corresponding SD values of the PRE and POST groups of samples were compared to one another and each to the values obtained in blood samples of cancer-free subjects (CON) and in patients with hematological malignancies taken at diagnosis (ADI). The SD values for *TP53*, *AML1*, and *CEN17 *in the POST group were low, similar to those of the CON group. Yet, each of the three tested loci in the PRE group showed high SD values, similar to those observed in the ADI group (solid bars in Figure 2; Columns 1-3 in Table 1). The differences in the SD values between the POST and PRE groups were highly significant, P < 10^-6 ^for *TP53 *and *AML1*, and P < 10^-7 ^for *CEN17 *(Table 1). The mean SD values for *TP53, CEN17 *and *AML1 *in the POST group were similar to those in the CON group, but were almost half of those found in the PRE and ADI groups. Each SD value in the POST samples exhibited highly significant differences from the corresponding values in the PRE and ADI groups (Table 1).

**Table 1:** Levels of significance (P) of the differences with respect to SD values and aneuploidy levels.

	*TP53*	*CEN17*	*AML1*	Chromosome 17	Chromosome 21
CON Vs. ADI	P < 10^-15^	P < 10^-14^	P < 10^-18^	P < 10^-6^	P < 10^-13^

CON Vs. PRE	P < 10^-9^	P < 10^-12^	P < 10^-6^	P < 10^-7^	P < 10^-4^

CON Vs. POST	P > 0.25	P > 0.25	P > 0.85	P < 10^-10^	P < 10^-5^

POST Vs. ADI	P < 10^-7^	P < 10^-8^	P < 10^-8^	P > 0.55	P > 0.05

POST Vs. PRE	P < 10^-6^	P < 10^-7^	P < 10^-6^	P > 0.10	P > 0.20

ADI Vs. PRE	P > 0.10	P > 0.35	P > 0.5	P > 0.05	P < 0.002

CON - samples of cancer free subjects (20 cases); ADI - samples of patients with hematological malignancies obtained at diagnosis (20 cases); PRE - samples of patients with hematological malignancies taken prior to alloSCT (14 cases); POST - samples of patients with hematological malignancies taken following alloSCT (10 cases); SD values - columns 1-3; and aneuploidy levels - columns 4 and 5.

The SD values for *TP53, AML1 *and *CEN17 *are normally significantly lower (as exemplified by the CON group) than those of an imprinted locus (exemplified here by the *SNRPN *gene) (Figure 2A; Table 2, first row). However, blood samples of the PRE group (as well as the ADI group) had high SD values, similar to those characterizing an imprinted locus (Table 2; solid bars in Figure 2). Yet, following transplantation, i.e., in the cell samples of the POST subgroup, the SD values of the tested loci were set to normal and as such deviated significantly (P < 10^-7^; Table 2, last row) from those of the imprinted *SNRPN*.

**Table 2:** Levels of significance (P) of the differences between the SD values for the designated loci in respect to those of *SNRPN*.

	TP53	CEN17	AML1
CON (n = 20)	P < 10^-10^	P < 10^-10^	P < 10^-10^

ADI (n = 20)	P > 0.55	P > 0.65	P < 0.03

PRE (n = 14)	P > 0.40	P > 0.75	P > 0.95

POST (n = 10)	P < 10^-7^	P < 10^-7^	P < 10^-7^

CON - samples of cancer free subjects; ADI - samples of patients with hematological malignancies obtained at diagnosis; PRE - samples of patients with hematological malignancies taken prior to alloSCT; POST - samples of patients with hematological malignancies taken following alloSCT; and n - Number of cases tested within each group for each locus. Each value (for each designated locus in each group of samples) is in respect to the levels of SD for *SNRPN *in the blood samples of the CON group (20 cases). The SD level for *SNRPN *in the ADI group was similar (P > 0.70) to that obtained in the CON group (see last bars in Figure 1A and 1B).

Thus the three independent genetic loci under study in patients with hematological malignancies show abnormal replication properties (loss of synchrony), but following alloSCT, all three regain their normal inherent replication mode (solid bars in Figure 2; Tables 1 and 2).

### Following stem cell transplantation malignancy-associated aneuploidy is unchanged

**Figure 3:**
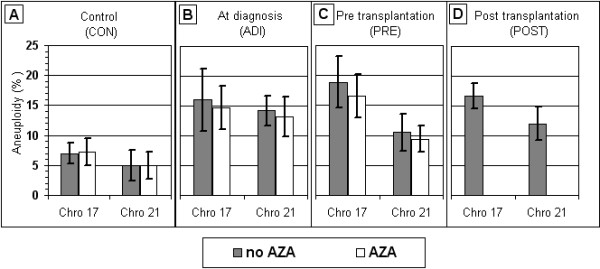
**Mean frequency of aneuploidic cells in blood samples grown with and without AZA**. (A) Control samples (20 cases); (B) samples of patients with hematological malignancies obtained at diagnosis (20 cases); (C) samples of patients with malignancies taken prior to alloSCT transplantation (14 cases); and (D) samples of patients with haematological malignancies taken following alloSCT (10 cases). Chro - Chromosome; and AZA - 5-azacytidine.

Applying FISH using the *CEN17 *and *AML1 *probes (enabling the enumeration of chromosomes 17 and 21); we examined the numbers of these chromosomes at interphase in blood cell samples of the four groups of subjects under study. The mean and standard deviations of the frequency of cells showing aneuploidy, changes in the normal copy number for the POST group was 16.8 ± 2.2% (ranging from 13.5-20.5%) for chromosome 17, and 12.0 ± 2.8% (ranging from 8.0-16.5%) for chromosome 21. These values appear to be significantly higher (P < 10^-10 ^and P < 10^-5 ^for chromosomes 17 and 21, respectively) than the corresponding values obtained in the CON group, ranging from 4.0-10.5% for chromosome 17, and 0.5-9.0% for chromosome 21, with mean values of 7.0 ± 1.7% and 5.0 ± 2.6%, respectively (solid bars in Figure 3). In contrast, no differences were found in the frequency of cells with aneuploidy, for the two chromosomes under test, between cells of the POST group and those of each of the other two groups of patients with hematological malignancies (solid bars in Figure 3; Columns 4 and 5 in Table 1).

The high level of aneuploidy in the POST group of blood samples was manifested both with regard to monosomy (a loss of one chromosomal copy) as well as multisomy (a gain of one or more chromosomal copies) for each of the two chromosomes tested. For each chromosome, the monosomy as well as the multisomy values, in the cells of the POST group were similar to the corresponding values in the PRE and ADI groups and significantly higher than those observed in the CON group (data not shown).

**Figure 4:**
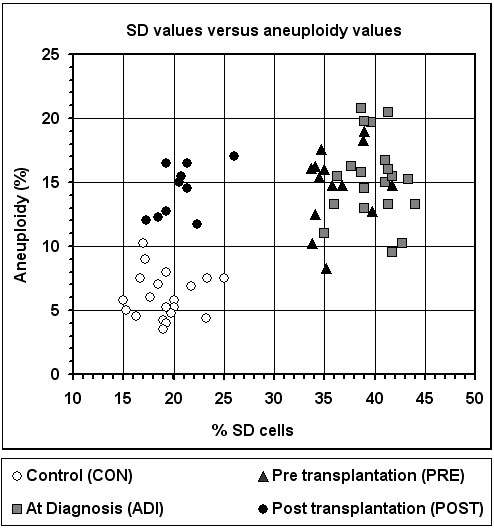
**Distribution of blood samples according to their SD cell frequency and aneuploidy level**. The SD value of each sample is expressed by the mean of the SD cell frequency values of *TP53*, *CEN17*, and *AML1*; and the aneuploidy is the mean of the aneuploidy values of chromosomes 17 & 21.

Finally, when all of the 64 blood cell samples studied are compared with respect to two factors -- (i) the mean SD values for *TP53*, *CEN17 *and *AML1*; and (ii) the mean aneuploidy value of chromosomes 17 and 21 -- it is clearly seen that the cancer-related aberrant SD value (an epigenetic marker) is set back to the normal following transplantation, while the cancer-associated increase in aneuploidy level (a genetic marker) is not readjusted to the background level (Figure 4).

Clearly, the loss of synchrony as well as the elevated aneuploidy in the patients' cells was neither type-of-malignancy specific nor phase-of-malignancy specific. This is evident from the wide consistency in the values within the PRE group, representing various hematological malignancies at different stages of severity, including remission (1 ALL and 3 AML cases), chronic (6 CML cases) and accelerated (1 NHL, 1 AML and 2 CML cases) (Figure 4). Similarly, within the ADI group (2 ALL, 9 AML, 8 CML and 1 MM cases) a consistency in the replication and aneuploidy values was observed in the various conditions (Figure 4).

### The cancer-related asynchronous replication is revealed in bone marrow cells

**Figure 5:**
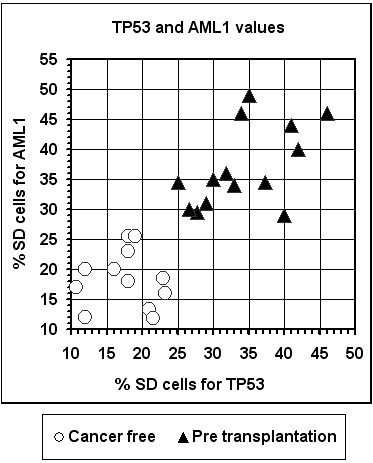
**Distribution of bone marrow samples according to their *AML1 *and *TP53 *SD values**.

Bone marrow cells of the PRE group of patients showed high SD frequencies for *TP53 *and *AML1*, similar to those in the patients' blood samples, yet significantly higher (P < 10^-7 ^and P < 10^-8^, for *TP53 *and *AML1*, respectively) than those from bone marrow cells of cancer-free individuals. Specifically, the frequency of SD cells in bone marrow samples of the pre transplantation patients ranged from 25.0-46.0% and 29.0-49.0% for *TP53 *and *AML1*, respectively, whereas the corresponding values for cancer free individuals ranged respectively from 10.7-23.2% and 12.0-25.5% (Figure 5).

Thus, the bone marrow results indicate that the cancer-related replication aberration, shown previously in blood cells (Figure 2C), is revealed also in the bone marrow cells and it further shows that the loss of synchrony is non-specific to the type or stage of the hematological disease. The bone marrow samples derived from pre transplantation patients differing in the type and stage of malignancy all displayed similar (high) SD values for each gene studied, which differed from the normal values characterizing bone marrow cells of healthy individuals (Figure 5).

### Asynchronous replication is not observed in lymphocytes of cancer-free individuals with chronic prostate inflammation

**Figure 6:**
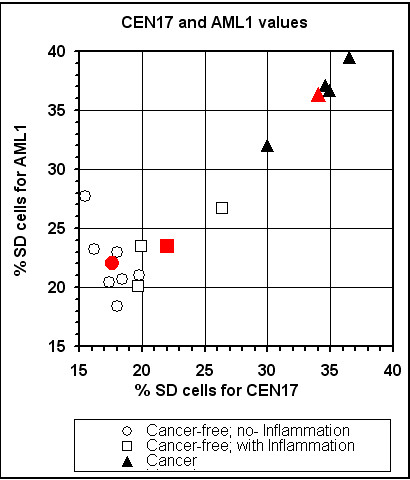
**Distribution of lymphocyte samples of patients with prostate cancer and cancer-free individuals, with and without inflammation of the prostate gland, according to their *AML1 *and *CEN17 *SD values**. The red marked characters indicate the mean values of the corresponding subgroup; Note, that the SD values of samples derived from the cancer-free patients with chronic inflammation were similar to those of the cancer-free patients with no inflammation, and were lower than those found in the cancer patients.

In a related study, we have examined the frequency of SD cells of two loci (*AML1 *and CEN17) in blood cell samples of three subgroups of urological male patients: (i) patients with prostate cancer (four cases); (ii) cancer-free patients suffering from prostate chronic inflammation (three cases); and (iii) matching cancer-free patients with benign prostate hyperplasia, who were not found to suffer from infectious disease (seven cases). This study was performed, in part, to see whether the high SDs of patients with malignancies were not simply a result of inflammation. The SD values of samples derived from the cancer-free patients with chronic inflammation were similar to those of the cancer-free patients with no inflammation, and were lower than those found in the cancer patients (Figure 6).

### 5-azacytidine (AZA) mimics the effect of stem cell transplantation -- rehabilitation of the epigenetic markers but not the genetic ones

Blood cell samples of the PRE and the ADI groups grown in the presence of AZA, showed a low frequency of SD cells for *TP53, AML1 *and *CEN17*, similar to those observed in the cell samples of the CON and the POST groups (Figure 2; Columns 1-3 in Table 3). Yet, the increased frequencies of aneuploidy shown in the PRE and ADI samples did not return to normal in the presence of AZA (Figure 3; columns 5 and 6 in Table 3). In this respect, AZA is mirroring the long term effect of G-CSF, as shown in peripheral blood lymphocytes of healthy stem cell donors following in-vivo application of the stimulating factor -- Rehabilitation of the replication pattern and fixation of the elevated aneuploidy levels [21].

**Table 3:** Levels of significance (P) of the differences between corresponding values in samples grown with and without AZA.

	TP53	CEN17	AML1	SNRPN	Chromosome 17	Chromosome 21
CON (n = 20)	P > 0.20	P > 0.10	P > 0.05	P > 0.70	P > 0.60	P > 0.50

ADI (n = 20)	P < 10^-13^	P < 10^-12^	P < 10^-15^	P > 0.50	P > 0.30	P > 0.20

PRE (n = 14)	P < 10^-11^	P < 10^-9^	P < 10^-6^	-----	P > 0.10	P > 0.40

CON - samples of cancer free subjects; ADI - samples of patients with hematological malignancies obtained at diagnosis; PRE - samples of patients with hematological malignancies taken prior to alloSCT; AZA - 5-azacytidine; n - Number of cases tested within each group for each locus/chromosome; SD values - columns 1-4; and aneuploidy levels - columns 4 and 5.

It should be noted that there were no changes in the high SD values for *SNRPN *of samples grown in the presence and the absence of AZA as shown in the cell samples of the CON group (mean values of 38.6 ± 5.7 and 37.9 ± 7.0, respectively) as well as in those of the ADI group (mean values of 37.7 ± 3.6 and 38.1 ± 5.0, respectively) (Figure 2A and 2B; Table 3). Thus, it appears that AZA abolishes only asynchronous replication that arises from the malignant condition but not that normally hatched in the genome.

## Discussion

We investigated the temporal order of allelic replication of two cancer-related genes (*TP53 *and *AML1*) and a non-coding repetitive DNA sequence (*CEN17*): *TP53 *is a tumor-suppressor gene, the most commonly mutated gene in human neoplasms (reviewed in [22]); *AML1 *is an *ETS *family gene, involved in the 8;21 -- leukemia-abundant translocations (reviewed in [23]); and *CEN17 *is an array associated with the centromere of chromosome 17, which, as such, is engaged in chromosome segregation [18]. Normally, these loci exhibit a synchronous mode of allelic replication. Yet in peripheral blood cells of blood cancer patients prior to transplantation, each displayed loss of synchrony and replicated asynchronously, a behavior normally observed in imprinted genes (reviewed in the introduction). This aberrant replication seen in these patients appears to be non-disease and non-disease-status-specific as we showed that it characterizes various hematological malignancies and it was displayed both at diagnosis as well as at advanced stages of the disease. It was observed at remission and at the relapse of the basic malignancy. These results are in line with previous studies reporting that blood cells of patients with hematological malignancies taken at diagnosis exhibit a loss of synchrony of normally biallelically expressed genes [16,21].

Furthermore, we could use the cancer related replication marker in bone marrow cell samples to differentiate between patients prior to transplantation and control subjects, thereby showing that in hematological malignancies the epigenetic insult is not specific to blood cells, and it is displayed in bone marrow cells of patients undergoing treatment, shown here, as well as at diagnosis, shown previously [15].

However, this abnormal pattern of replication (an epigenetic aberration), which is shown to be independent of disease status, is abolished in lymphocytes derived from patients following allogeneic stem-cell transplantation. Yet, the increased aneuploidy level (a genetic aberration) that accompanies hematological malignancies (shown in blood cells of patients at diagnosis as well as prior to transplantation), unlike the epigenetic aberration, is not reduced to normal following transplantation.

Interestingly, the effect of alloSCT, in respect to replication adjustment and aneuploidy maladjustment, resembles the long term outcome of granulocyte colony stimulating factor (G-CSF) treatment displayed in lymphocytes of healthy stem-cell donors [21]. Briefly, the aberrant replication in cells of the donors gradually, in the course of several weeks after G-CSF administration, readjusts to normal, while the increased level of sporadic aneuploidy remains unchanged. The finding that blood lymphocytes of hematological patients following alloSCT exhibit replication and aneuploidic properties similar to those of the G-CSF treated healthy donors [21] is not surprising. The blood cells of patients following alloSCT are actually donor cells that were exposed to G-CSF administered prior to collection to boost stem cell release.

Whatever the reason, we show here that the aberrant replication patterns characterizing blood cells derived from patients with hematological malignancies is completely eradicated following a successful therapeutic modality, i.e., alloSCT. As such, this test may find use as a convenient indicator of full biological recovery and cure. Hence, the level of asynchrony of a biallelically expressed locus compared to that of an imprinted (monoallelic) locus offers a potential epigenetic marker for assessing the outcome of transplantation. It would allow simpler determinations of the success of alloSCT in biologically eradicating the baseline malignancy and yielding a complete cure, a determination now requiring laborious, costly techniques [24].

Yet, the increased aneuploidy level (a genetic aberration), unlike an epigenetic aberration, is not reversible. This is probably corollary to the critical difference, even on the cellular level, between an epigenetic aberration and a genetic one; the former is potentially reversible and the latter is not. This difference enables medicine to take advantage of cancer-induced epigenetic alterations for disease control and treatment follow up. As such the irreversible nature of aneuploidy explains why the aneuploidy levels are not reduced (during the course of time) in blood cells of the donors as well as in those derived from the recipients. However, it is encouraging that in both cases they are not elevated and remain the same (steady state). This is because, from a cytogenetic point of view, a "steady state" of aneuploidy (even at elevated levels) in contrast to continuous increase in aneuploidy levels (chromosomal instability) is considered harmless [25]. This is in line with the view that G-CSF applied to healthy donors is safe [26,27].

Finally, we show here that a demethylating anti-cancer drug (5-azacytidine), applied in vitro mimics the recuperating effect of transplantation, eradiating the epigenetic aberration, while leaving the genetic aberration as is. First, our results indicate a clinical potential for the use of the replication marker, particularly for drug monitoring. Second, they further ease the theoretical concerns that healthy donors are at risk by their being given G-CSF, but agree with data indicating that healthy donors following G-CSF administration display alterations in their gene expression patterns [[28]; see also the next paragraph]. Third, they link the aberrant replication with enhanced methylation capacity. The latter was expected, as numerous studies have indicated that the epigenetic disequilibrium accompanying the cancerous phenotype is associated with global and specific methylation modifications [29-31]. Methylation modifications were also described in blood and cytologically normal cells adjacent to solid tumors [32-35].

Yet, allelic counterparts that normally replicate asynchronously are usually differentially methylated [reviewed in [3]]. Therefore it is reasonable to assume that the malignancy disrupts the epigenetic symmetry of alleles achieved during diploidization. This correlates with the epigenetic gene silencing known to accompany the cancerous status [29]. Furthermore, there is evidence that epigenetic gene silencing in cancer is not a localized event affecting discrete genes, but a generalized condition that spans broad chromosomal regions [36]. This is in accord with our results showing that both the tumor-suppressor *TP53 *gene -- a normally biallelically expressed gene located at band p13.1 of chromosome 17 -- and the non-coding pericentromeric region of chromosome 17 (*CEN17*) display epigenetic alterations in the patients' cells.

Interestingly, the demethylating anti-cancer drug AZA eradicated the aberrant asynchronous replication in the cells we studied, but had no effect on the asynchronous replication characterizing a normal imprinted gene. This is in accord with studies showing that the AZA drug and its deoxy-derivative (5-aza-2'-deoxycytidine) have profound effects on abnormal cells and more moderate interactions with normal ones. Both these drugs reactivate the expression of genes that have undergone epigenetic silencing, particularly when this silencing occurred in a pathological situation (reviewed in [37]). The two nucleoside analogues (5-azacytidine and 5-aza-2'-deoxycytidine) are converted to the deoxy-nucleotide triphosphates, which may replace cytosine during DNA replication. As such, they are active only in S-phase [38]. It would be interesting to determine whether other demethylating anti-cancer drugs also abolish aberrant asynchronous replication exhibited by biallelically expressed genes associate with pathological situations while disregarding asynchronous replications hatched normally in monoallelically expressed genes. In this context, it is worthy to note that the wide spread of epigenetic aberrations in neoplasmic cells [29-31], coupled with the reversible nature of the epigenetic changes, opens a new therapeutic avenue for the development of new anti-cancer drugs capable of reversing such aberrant epigenetic traits (reviewed in [31]). However, any promising anti-cancer drug, based on this epigenetic therapeutic approach, must be able to differentiate between the cancer-induced and normal (inherent) allelic asynchronous replication.

## Conclusions

Patients with hematological malignancies display non-disease-specific epigenetic and genetic aberrations in their blood cells. These aberrations, detected by simple molecular-cytogenetic means are displayed in the patients' cells from the time of diagnosis through to just before they undergo allogeneic stem cell transplantation (alloSCT). Thereafter, concomitantly with full remission, the aberrant epigenetic markers are completely eradicated, leaving the genetic markers unchanged. In fact, blood cells derived from patients following successful alloSCT mirror the long-term effects displayed by blood cells of healthy stem cell donors following administration of granulocyte colony stimulating factor (G-CSF). Hence, the donor's cells in the recipient's "environment" behave as they do in their own "environment." An archetypical demethylating drug, 5-azacytidine (AZA), which has recently been approved for the therapy of myeloid malignancies, mimics the effect of alloSCT; AZA applied in vitro to blood cells of patients prior to alloSCT eradicates the epigenetic aberrations, leaving unchanged the genetic ones. Our results, on one hand, ease the theoretical concern about the safety of G-CSF administration to healthy stem-cell donors and on the other hand, offer a potential new approach for evaluating the success of transplant or other treatments for blood cancers, and for long-term follow up of the patients who have overcome a hematological malignancy.

## Competing interests

MM and LA are cofounders of Allelis Diagnostics Ltd. All other authors declare that they have no competing interests.

## Authors' contributions

AN conceived the study, collected most of the samples of the patients with hematological malignancies and participated in drafting the manuscript. AK-I carried out the experimental work and participated in sample collection. SC and MM designed and performed the part of the study dealing with the urological patients. LA was responsible for the overall conception of the study, analyzing the results and drafting the manuscript. All authors read and approved the final manuscript.

## Pre-publication history

The pre-publication history for this paper can be accessed here:

http://www.biomedcentral.com/1471-2407/10/230/prepub
